# Disparities in access to food and chronic obstructive pulmonary disease (COPD)-related outcomes: a cross-sectional analysis

**DOI:** 10.1186/s12890-021-01485-8

**Published:** 2021-04-27

**Authors:** Eric Moughames, Han Woo, Panagis Galiatsatos, Karina Romero-Rivero, Sarath Raju, Vickram Tejwani, Eric A. Hoffman, Alejandro P. Comellas, Victor E. Ortega, Trisha Parekh, Jerry A. Krishnan, Michael B. Drummond, David Couper, Russell G. Buhr, Robert Paine, Joel D. Kaufman, Laura M. Paulin, Nirupama Putcha, Nadia N. Hansel

**Affiliations:** 1 Department of Medicine, Johns Hopkins University, 4940 Eastern Avenue, Baltimore, MD 21224 USA; 2 Division of Pulmonary and Critical Care, Johns Hopkins University, 4940 Eastern Avenue, Baltimore, MD 21224 USA; 3 Department of Internal Medicine, University of Iowa, Iowa City, IA USA; 4 Department of Medicine, Wake Forest School of Medicine, Winston Salem, NC USA; 5 Department of Medicine, University of Alabama, Birmingham, AL USA; 6 Department of Medicine, University of Illinois, Chicago, IL USA; 7 Department of Medicine, University of North Carolina At Chapel Hill, Chapel Hill, NC USA; 8 Department of Biostatistics, University of North Carolina At Chapel Hill, Chapel Hill, NC USA; 9 Department of Medicine, Greater Los Angeles Veterans Affairs Healthcare System, Los Angeles, CA USA; 10 Department of Medicine, University of Utah, Salt Lake City, UT USA; 11 Department of Medicine and Epidemiology, University of Washington, Seattle, WA USA; 12 Department of Medicine, Dartmouth Hitchcock Medical Center, Lebanon, NH USA

**Keywords:** Food, Access, Food desert, Disparities, COPD, Respiratory

## Abstract

**Background:**

Millions of Americans are living in food deserts in the United States, however the role of the local food environment on COPD has not been studied. The aim of this study is to determine the association between food deserts and COPD-related outcomes.

**Method:**

In this cross-sectional analysis we linked data collected from SPIROMICS (SubPopulations and InteRmediate Outcome Measures in COPD Study) between 2010 and 2015 and food desert data, defined as an underserved area that lacks access to affordable healthy foods, from the Food Access Research Atlas. COPD outcomes include percentage of predicted forced expiratory volume in one second (FEV1%), St. George’s Respiratory Questionnaire (SGRQ), COPD Assessment Test (CAT), 6-min walk distance test (6MWD), exacerbations, and air trapping. We used generalized linear mixed models to evaluate the association between living in food deserts and respiratory outcomes, adjusting for age, gender, race, education, income, marital status, BMI, smoking status, pack years, and urban status

**Results:**

Among 2713 participants, 22% lived in food deserts. Participants living in food deserts were less likely to be white and more likely to have a lower income than those who did not live in food deserts. In the adjusted model controlling for demographics and individual income, living in food deserts was associated lower FEV1% (β = – 2.51, *P* = 0.046), higher air trapping (β = 2.47, *P* = 0.008), worse SGRQ (β = 3.48, *P* = 0.001) and CAT (β = 1.20, *P* = 0.003) scores, and 56% greater odds of severe exacerbations (*P* = 0.004). Results were consistent when looking at food access alone, regardless of whether participants lived in low income areas.

**Conclusions:**

Findings suggest an independent association between food desert and food access alone with COPD outcomes. Health program planning may benefit from addressing disparities in access to food.

**Supplementary information:**

The online version contains supplementary material available at 10.1186/s12890-021-01485-8.

## Introduction

Chronic obstructive pulmonary disease (COPD) is a leading cause of morbidity and mortality in the United States (U.S.) and the world, with almost 6% of the population in the United States having COPD [1–3]. Epidemiologic studies have highlighted socioeconomic and environmental factors as major contributors to COPD prevalence and adverse health outcomes [4–9]. Poor dietary intake has been attributed to worse COPD outcomes [10, 11]. Further, several studies have investigated the association of food deserts, defined as underserved areas that lack access to affordable healthy foods, with health outcomes [12–16]; however, the association between food desert and COPD has not yet been established. Further, it is empirically difficult to distinguish whether these associations are due to low socioeconomic status (SES) factors or low food access; that is, areas where more than one-third of the population have limited access to the nearest supermarket [12–16]. Meaningful differences in food access exist in neighborhoods with similar SES, such that low food access is not strictly found in low income areas, but rather both low and high food access areas exist within high and low SES neighborhoods [17, 18], and the effect of food access alone on health has been studied seldom, with mixed results [19–23]. No currently published studies have investigated the association between food desert, nor low food access areas, and COPD outcomes.

Given the importance of diet on COPD outcomes and lack of studies investigating the association between food desert and COPD, the present work links a nationally-available food access dataset to the SPIROMICS Air (SubPopulations and InteRmediate Outcome Measures in COPD Study) dataset to evaluate the association of living in food desert and low food access areas on COPD-related respiratory outcomes, and further investigate whether these associations differ by neighborhood poverty or urban–rural status. We hypothesize that living in a food desert area, and in particular living in an area with low food access, is associated with worse COPD outcomes.

## Methods

The SPIROMICS study is a multicenter study that includes current and former smokers (≥ 20 pack years) with or without airflow obstruction and healthy participants who were non-smokers aged 40 to 80 years recruited from 12 clinical sites spread across the United States. Only participants who were current or former smokers were analyzed here [24]. Particpants who were missing 2010 census geographic identifiers, food desert data, or who did not consent for geocoding, were excluded (Additional file 2: Appendix Figure E1). The Institutional Review Board of Columbia University, Johns Hopkins University, Wake Forest University, University of Utah, University of Michigan, University of California at Los Angeles, University of California at San Francisco, University of North Carolina at Chapel Hill, National Jewish Health, University of Illioins at Chicago, University of Alabama, Temple University and University of Iowa along with all other institutions that were participating in SPIROMICS approved the study protocols and granted ethical approval. Informed consent was obtained from all subjects.

### Assessment of food desert and food access

As part of SPIROMICS AIR [25], an ancillary study to SPIROMICS with the goal of understanding the effects of air pollutants on COPD outcomes, census tract data of participants’ residential addresses was linked to the Food Access Research Atlas (FARA), a web-based mapping tool from the United States Department of Agriculture’s Economic Research Service [12]. The food desert data was from year 2010; SPIROMICS AIR data was collected between 2010 and 2015. Food desert was defined as an area of low income and low food access [12]. Low food access area was defined as an area where at least a third of people live more than 0.5 miles in urban areas or more than 10 miles in rural areas from the nearest supermarket, supercenter or large grocery stores [12], an approach utilized in several other research studies [19–21, 26–29]. Low income area was defined as an area where the poverty rate is 20 percent or greater, or the area’s median family income is less than or equal to 80% of the state-wide median family income, or is in a metropolitan area and has a median family income less than or equal to 80% of the metropolitan area's median family income [12].

## Outcomes

COPD related outcomes included baseline percentage of predicted forced expiratory volume in one second (FEV_1_% predicted) [30], respiratory health-related quality of life using the St. George’s Respiratory Questionnaire (SGRQ) [31], COPD health status using COPD Assessment Test (CAT) scores [32], dyspnea using modified Medical Research Council (mMRC) dyspnea scale [33], exercise capacity using the 6-min walk distance test (6MWD) [34], and exacerbations (moderate and severe, where moderate exacerbations were defined as worsening respiratory symptoms requiring additional medication and an urgent care or unscheduled physician visit, and severe exacerbations were defined as worsening respiratory symptoms requiring hospitalization or an emergency department visit) in the last 12 months. Airway structure using CT- related outcomes included total % emphysema, and air trapping defined as the percentage of lung voxels in the field below − 856 Hounsfield units at residual volume [35].

### Covariates

Covariates include age, gender, self reported race (white vs. non-white), education (some college or above vs. less than some college), income (under $15,000, $15,000-$34,999, $35,000–49,999, $50,000–74,999, above $75,000, declines to answer/missing), marital status (married vs. not married), BMI (continuous), smoking status (current smokers vs. former smokers), pack years, and urban status (urban vs. rural, where urban and rural status was defined at the census tract level for each participant based on established criteria from the U.S Census Bureau, which classifies each census tract as either urban or rural depending on population density) [36]. In addition, a categorical variable for clinical sites was included as a covariate.

Other covariates for the food access results included neighborhood poverty, a dichotomous variable, indicating low income tract vs. non-low income tract, merged directly from Food Access Research Atlas (FARA) [12]. This variable, along with food access, constitute the official definition of food desert as stated previously.

### Statistical analysis

Descriptive analysis was performed of all variables using summary statistics, histograms and scatter plots. Participant characteristics were compared for those who are residing in food deserts versus non-food deserts as well as low food access versus non-low food access, and their differences were tested using t-tests and chi-squared tests. To assess differences in COPD-related outcomes across food deserts and low food access areas, cross-sectional linear and logistic regression was performed for each continuous and dichotomous COPD outcome on food desert or food access adjusting for covariates. We tested for multicollinearity by computing variance inflation factor (VIF), and all predictors had VIF < 4 across different models. As a sensitivity analysis, the models were run adjusting for FEV1% predicted; and as secondary analyses, to determine whether differences in food desert or access on respiratory outcomes differed by COPD status, interaction terms were tested and stratified models by COPD status were conducted.

Based on a priori hypotheses, the effect modification by urban status and neighborhood poverty on food access’s association with COPD-outcomes was examined. A multiplicative interaction term between food access and either urban status or neighborhood poverty was included, adjusting for the remaining covariates. Direction and statistical significance of interaction was assessed. The effect estimates of food access within urban and rural or low income and non-low income tracts were also separately estimated using stratified analysis. The effect modification by urban status on food desert was not tested because of the limited sample size (n = 10) for participants residing in rural areas that were food deserts.

All statistical analyses were performed using STATA version 15.1. The threshold of *P* < 0.05 was used for statistical significance for main and interaction effects.

## Results

### Participant characteristics

Current or former smokers (n = 2713) (Table 1) were included and they were 46% female, 77% white with a mean age of 63.5. The majority of participants (56%) had at least some college education and half (52%) had an annual income under $50,000. Almost a quarter (22.2%) of participants resided in food desert areas and were less likely to be white or married; and more likely to be female, have lower educational attainment and an income under $15,000 compared to those that do not reside in food deserts (Table 1). In addition, participants residing in food desert areas were more likely to be current smokers compared to those who do not live in food desert areas. Of the participants living in food deserts 64% had COPD, and of those who did not live in food deserts 67% had COPD. (Table 1).

**Table 1:** Participant characteristics

	Food desert^a^ (N = 604)	Non-food desert^a^ (N = 2109)	*p* Value
FEV1% pred., mean ± SD	71.7 ± 27.2	73.4 ± 26.3	0.159
COPD (Strata 3 & 4)^b^, %	64.2	66.9	0.230
Age, mean ± SD	60.6 ± 8.8	64.3 ± 8.8	** < 0.001**
Female, %	50.2	44.9	**0.021**
White, %	61.8	80.9	** < 0.001**
Some college or above, %	44.9	59.4	** < 0.001**
Income, %			** < 0.001**
Under $15,000, %	30.8	17.2	
$15,000–$34,999, %	24.0	17.7	
$35,000–$49,999, %	11.5	12.7	
$50,000–$74,999, %	11.8	15.0	
> $75,000, %	8.0	19.5	
Decline to answer, %	13.8	18.0	
Married, %	35.6	50.0	** < 0.001**
Nonrural, %	98.3	86.2	** < 0.001**
BMI, %	28.3 ± 5.6	27.8 ± 5.2	0.064
Currently Smoking, %	54.1	36.0	** < 0.001**
Pack years, mean ± SD	47.6 ± 32.8	49.8 ± 25.0	0.082
Low income tract, %	100.0	23.1	** < 0.001**
Low food access, %	100.0	55.5	** < 0.001**

Data are given as percentages unless otherwise indicated

Bold values indicate statistical significance at the *p* < 0.05 level

^a^Food desert refers to areas defined as both low food access and low income tracts. Low food access refers to a census tract with at least 500 people or 33 percent of the population living more than 1/2 mile (urban areas) or 10 miles (rural areas) from the nearest supermarket, supercenter, or large grocery store. Low income tract refers to a census tract where the tract's poverty rate is 20 percent or greater, or the tract's median family income is less than or equal to 80% of the State-wide median family income, or the tract is in a metropolitan area and has a median family income less than or equal to 80 percent of the metropolitan area's median family income

^b^Stratum 3 includes participants with > 20 pack-years and mild/moderate COPD with FEV_1_ /FVC < 0.7 and FEV1 > 50% predicted (GOLD stage 1 and 2) measured during enrollment. Stratum 4 includes participants with > 20 pack-years and severe COPD with FEV_1_ /FVC < 0.7 and FEV_1_ < 50% predicted (GOLD stage 3 and 4) measured during enrollment [24]

There were an additional 1171 participants who also lived in low food access areas, but not in poverty areas. Those residing in low food access areas were more likely to be white, married, have a higher educational attainment and individual income, and more likely to be residing in urban areas than those residing in non-low food access areas (Additional file 3: Appendix Table E1).

## Multivariable regression

### Association of food deserts and COPD-outcomes

In minimal adjusted analyses adjusting only for study site, residing in food deserts was associated with worse quality of life and respiratory symptoms including worse CAT score, SGRQ score, shorter 6-MWD, and greater odds of exacerbations and mean % emphysema than those not residing in food desert areas (Table 2). In fully adjusted models controlling for demographics and individual SES, living in food deserts continued to be associated with several measures of COPD morbidity. Specifically, residing in a food desert was associated with 3.5 points higher SGRQ (β = 3.48, *P* = 0.001) and CAT (β = 1.20, *P* = 0.003) scores, and a shorter 6 MWD (β = − 12.7, *P* = 0.025). Additionally living in a food desert was associated with objective measures, including lower FEV1% predicted (β = − 2.51, *P* = 0.046) and higher air trapping (β = 2.47, *P* = 0.008) on CT imaging. Furthermore, participants residing in food deserts had 36% greater odds of any exacerbation (OR = 1.36, *P* = 0.010) and 56% greater odds of severe exacerbations (OR = 1.56, *P* = 0.004) in the prior 12 months than did those not living in food deserts (Table 2).

**Table 2:** Differences (95% CI) in COPD-related outcomes for former and current smokers in study population residing in food desert areas versus non-food desert areas

	Minimally adjusted^a^			Adjusted^b^		
	Mean difference or odds ratio (95% CI)		*P *value	Mean difference or odds ratio (95% CI)		*P *value
Lung function						
FEV%Pred	− 0.27	(− 2.70, 2.16)	0.828	− 2.51	(− 4.97, − 0.05)	**0.046**
COPD (OR)^c^	0.91	(0.74, 1.11)	0.333	1.20	(0.96, 1.50)	0.112
Quality of life/respiratory symptoms						
CAT	2.38	(1.58, 3.18)	** < 0.001**	1.20	(0.40, 2.00)	**0.003**
mMRC	0.13	(0.03, 0.22)	**0.012**	0.06	(− 0.04, 0.16)	0.210
SGRQ total	5.70	(3.71, 7.70)	** < 0.001**	3.48	(1.49, 5.47)	**0.001**
6-min walk distance (meters)	− 16.0	(− 27.3, − 4.73)	**0.005**	− 12.7	(− 23.9, − 1.58)	**0.025**
**Chest CT metric**						
% emphysema (− 950)	− 1.09	(− 2.07, − 0.12)	**0.028**	0.30	(− 0.58, 1.18)	0.508
% air trapping (− 856)	− 1.39	(− 3.46, 0.68)	0.187	2.47	(0.65, 4.29)	**0.008**
Exacerbations, last 12 months						
Any (OR)^c^	1.42	(1.14, 1.76)	**0.002**	1.36	(1.08, 1.72)	**0.010**
Severe (OR)^c^	1.70	(1.27, 2.26)	** < 0.001**	1.56	(1.15, 2.13)	**0.004**

Bold values indicate statistical significance at the *p* < 0.05 level

^a^Adjusted by clinical centers

^b^Adjusted for clinical centers, age, sex, race, education, income, marital status, rural status, BMI, smoking status, and pack years

^c^Coefficient represents odds ratio

In sensitivity analyses, inclusion of FEV1% predicted as a covariate attenuated some of the associations between food desert and COPD-outcomes (Additional file 1: Appendix Table E2). Adjusting for the confounders along with FEV1% predicted, food desert remained statistically significantly associated with worse CAT, SGRQ and increased severe exacerbation risk. In addition, there was no statistically significant interaction by COPD status; but for those outcomes for which the interaction approached nominal significance (e.g., 6MWD, odds of any exacerbation), the direction of interaction was such that adverse associations between food desert and outcomes were generally more adverse for the participants with COPD (vs. without COPD) (Additional file 1: Appendix Table E3).

### Association of food access and COPD-outcomes

In order to understand whether the associations between food desert and COPD outcomes are driven by low food access or neighborhood poverty, we separately explored whether low food access alone was associated with COPD outcomes. There was generally no evidence of effect modification by neighborhood poverty on the associations between food access and COPD-outcomes except for CAT (ßintx = 1.38, *P* = 0.042) and any exacerbation (ORintx = 1.70, *P* = 0.001)**,** both of which suggested more adverse association with low food access in low income tracts than in non-low income tracts. But, in stratified analysis, food access was not associated with either CAT or any exacerbations across low and non-low income tracts [difference in CAT score 0.89, *P* = 0.23 in low income areas and 0.25, *P* = 0.59 in non-low income areas; odds ratio of any exacerbation was 1.27, *P* = 0.22 in low income areas and 1.01, *P* = 0.94 in non-low income areas].

For the remaining outcomes, there was no significant interaction between food access and neighborhood poverty. In models adjusted for demographics, individual SES and neighborhood poverty, residing in a low food access area was associated with higher odds of COPD, lower lung function, and higher emphysema and gas trapping. In addition, low food access was associated with higher dyspnea (mMRC: β = 0.10, *P* = 0.030), and worse quality of life (SGRQ: β = 3.30, *P* < 0.001). Furthermore, participants residing in a low food access area had 52% greater odds of severe exacerbation (OR = 1.52, *P* = 0.012) than did those who did not live in a low food access area (Table 3).

**Table 3:** Differences (95% CI) in COPD-related outcomes for former and current smokers in study population residing in low food access areas versus non-low food access areas

	Adjusted^a^		
	Mean difference or odds ratio (95% CI)		*P *value
Lung function			
FEV%Pred	− 4.40	(− 6.78, − 2.03)	** < 0.001**
COPD (odds ratio)^‡^	1.42	(1.13, 1.78)	**0.002**
Quality of life/respiratory symptoms			
CAT	0.70	(− 0.03, 1.43)	0.060
mMRC	0.10	(0.01, 0.20)	**0.030**
SGRQ total	3.30	(1.52, 5.10)	** < 0.001**
6-min walk distance (meters)	− 3.03	(− 13.7, 7.59)	0.576
Chest CT metric			
% Emphysema (− 950)	1.22	(0.33, 2.12)	**0.007**
% air trapping (− 856)	3.23	(1.45, 5.01)	** < 0.001**
Exacerbations, last 12 months			
Any (OR)^b^	1.24	(0.98, 1.58)	0.072
Severe (OR)^b^	1.52	(1.10, 2.10)	**0.012**

Bold values indicate statistical significance at the *p* < 0.05 level

^a^Adjusted for neighborhood poverty, clinical centers, age, sex, race, education, income, marital status, rural status, BMI, smoking status, and pack years

^b^Coefficient represents odds ratio

### Association of food access and COPD-outcomes by urban/rural status

Though almost all subjects residing in food deserts were living in urban areas preventing evaluation of urban / rural differences, 73 participants (4.7%) of those living in low food access areas were also living in rural areas. There was evidence of effect modification by urban / rural status on the associations between food access and COPD-outcomes (Additional file 3: Appendix Figure E2). For all but the exacerbation outcomes, the interaction effect estimates were statistically significant, and the direction of interaction was such that adverse associations between residing in low food access areas and COPD-outcomes were consistently greater if the participants resided in urban areas than in rural areas. For those residing in urban areas, low food access was adversely associated with nearly all COPD-outcomes considered (Table 4). Specifically, urban low food access was associated with a worse FEV1% predicted (β = − 5.25, *P* < 0.001), greater odds of COPD (OR = 1.52, *P* = 0.001), worse dyspnea (mMRC: β = 0.16, *P* = 0.002), worse CAT (β = 1.05, *P* = 0.009) worse SGRQ total score (β = 4.28, *P* < 0.001) and greater odds of exacerbation (OR = 1.35, *P* = 0.028) and severe exacerbations (OR = 1.62, *P* = 0.011). CT scan findings were also significantly worse with a higher percent emphysema (β = 1.54, *P* = 0.002) and higher percent air trapping (β = 4.04, *P* < 0.001). The magnitudes of associations between low food access and COPD-outcome were consistently larger among those who resided in urban areas than for all participants.

**Table 4:** Differences (95% CI) in COPD-related outcomes for former and current smokers in study population residing in low food access areas versus non-low food access areas by urban status

	Rural (N = 302)			Urban (N = 2411)		
	Mean difference or odds ratio (95% CI)		*P *value	Mean difference or odds ratio (95% CI)		*P *value
Lung function						
FEV%Pred	2.38	(− 3.75, 8.51)	0.445	− 5.25	(− 7.84, − 2.66)	** < 0.001**
COPD (OR)^a^	0.77	(0.41, 1.44)	0.415	1.52	(1.20, 1.94)	** < 0.001**
Quality of life/respiratory symptoms						
CAT	− 1.66	(− 3.60, 0.29)	0.094	1.05	(0.26, 1.84)	**0.009**
mMRC	− 0.24	(− 0.50, 0.02)	0.070	0.16	(0.06, 0.26)	**0.002**
SGRQ total	− 3.96	(− 8.69, 0.78)	0.101	4.28	(2.36, 6.20)	** < 0.001**
6-min walk Distance (meters)	31.5	(5.13, 57.8)	**0.019**	− 8.93	(− 20.5, 2.68)	0.132
Chest CT metric						
% Emphysema (− 950)	− 0.99	(− 3.19, 1.21)	0.377	1.54	(0.55, 2.52)	**0.002**
% air trapping (− 856)	− 1.35	(− 6.30, 3.59)	0.591	4.04	(2.11, 5.97)	** < 0.001**
Exacerbations, last 12 months						
Any (odds ratio)^a^	0.70	(0.37, 1.33)	0.275	1.35	(1.03, 1.76)	**0.028**
Severe (odds ratio)^a^	0.80	(0.29, 2.17)	0.661	1.62	(1.12, 2.34)	**0.011**

All modes were adjusted for clinical centers, age, sex, race, education, income, marital status, BMI, smoking status, and pack years

Bold values indicate statistical significance at the *p* < 0.05 level

^a^Coefficient represents odds ratio

## Discussion

To our knowledge, there have been no previous studies investigating the association of food deserts and the impact of low food access on COPD outcomes. The main findings of our study are three-fold. First, living in a food desert area was independently associated with worse COPD outcomes. Another key finding was the consistency in our results with food access alone, regardless of neighborhood SES, suggesting that limited food access is detrimental in both low and high income neighborhoods. Additionally, the connections between low food access and COPD outcomes were stronger in urban areas compared with rural areas.

There is increasing recognition that the neighborhood environment can impact chronic disease risk and outcomes [37–39]. In fact, studies have shown that living in food deserts has been associated with adverse outcomes focusing on obesity, diabetes and heart diseases [19–22, 40, 41]. In this current study, we show that living in a food desert area is also associated with several worse COPD outcomes, including lower lung function, higher odds of COPD exacerbations, and worse quality of life and CT evidence of gas trapping, even after adjusting for smoking status and individual income. Food desert is a composite measure of low food access and neighborhood poverty [12]. Low individual SES has been a recognized risk factor for poor COPD outcomes [42] and additional evidence is suggesting that neighborhood SES is also associated with worse outcomes [37–39]. Other factors imbedded within the neighborhood SES, such as higher air pollutant exposures, may also have a variety of influences on respiratory morbidity that could further explain our findings [38, 39].

When looking at food access alone, only a few studies have reported the association between availability of food stores and outcomes in chronic health conditions [20, 21, 37, 38]. Though results have been inconsistent, it has been demonstrated that the availability of supermarkets plays an important role in diet quality, is positively associated with a healthier food choice [40, 43–45], and is associated with lower prevalence of obesity and overweight [40]. Importantly, there are several studies suggesting that a healthier diet leads to better COPD outcomes [46, 47] perhaps by protecting the lungs from oxidative damage [47, 48]. For example, higher fruit and vegetable consumption and a lower intake of dairy products, red meats, sweets, and sugary drinks have been linked to lower prevalence of COPD, higher FEV1% predicted, and lower mortality in individuals with COPD; [46, 47, 49, 50] and omega 3 intake has been associated with improved COPD outcomes [51]. Therefore, it is reasonable to consider that access to local healthy foods may influence COPD respiratory outcomes [52]. In fact our results were consistent with these findings, demonstrating that when looking at food access alone, living in low food access areas was associated with worse COPD outcomes, specifically worse FEV1% predicted, a higher odds of COPD, worse CT imaging metrics including higher percent emphysema and air trapping and more severe COPD exacerbations. Further, the effect on COPD outcomes was largely similar whether individuals resided in a low SES neighborhood or a more affluent neighborhood. Only the association of low food access with CAT score and any exacerbation risk, was increased in areas that were also high poverty compared to lower poverty areas.

The results further suggest that the impact of low food access may be greatest in urban areas. Our results could be explained by the ubiquitous prevalence of unhealthy options (e.g., corner stores, fast food chains, etc.) in cities compared with rural areas [29], and in this type of urban setting, people will be more inclined to go to a fast food restaurant or corner store that is much closer and cheaper rather than walking farther away to a healthy food source [53–55]. As an example, a study in Austin, Texas, showed that there are three times more unhealthy food stores than healthy ones that are within walking distance in the city proper [29]. Additionally, people living in urban areas tend to pay significantly more, an estimated 3–37% more, than those who live in non-urban areas to purchase the same healthy groceries [56, 57].This may further discourage the urban community from purchasing healthy options, thus leading to worse COPD outcomes.

The study has several limitations. First, though we looked at the association of both food desert and food access adjusting for neighborhood poverty, there are additional neighborhood factors such as availability of pharmacy or health care that may track with food deserts and low food access and be confounding factors associated with worse COPD morbidity. Second, there are additional factors that contribute to healthy food choices in addition to access to healthy foods such as vehicle availability and personal choice [58]. This additional information was not available, nor was information on actual dietary intake, though the finding that low food access is associated with adverse outcomes in both lower and higher SES neighborhoods suggests that dietary factors may be playing a role. Further, the findings suggest a potential mediatory role of lung function in the relationship between food desert and COPD-outcomes. In addition, only a small number of participants lived in a rural low food access setting compared with urban areas. This restricts the power to identify an association between food access and COPD outcomes in rural areas. Given the high prevalence of COPD and high COPD morbidity in rural areas, the role of food desert and other contextual factors that may drive COPD morbidity in rural areas deserves further attention [59]. Also, our participants were all current or former smokers, thus we are unable to draw conclusions regarding association of residing in food desert or low food access areas with COPD outcomes among never smokers. Finally, the cross-sectional study design makes it difficult to explicitly infer causal links between food access and COPD outcomes.

In conclusion, in this multicenter study, we have demonstrated an independent association between food desert and worse COPD-related outcomes, but also the independent effect of food access alone on COPD outcomes, emphasizing the importance that diet and access to healthy food choices may have on respiratory morbidity. Associations persisted across varying degrees of individual- and contextual-level (i.e., neighborhood) factors supporting that low food access is problematic regardless of neighborhood income. Low food access results were more pronounced in urban compared to rural environments. Such insight warrants a further investigation of how COPD-related outcomes are impacted by the proximity to food access. Further, food access should be considered for utilization in novel and equitable health strategies in policy implications for local municipalities, in addition to current COPD-related clinical guidelines.

## Supplementary information

**Additional file 1.** Additional patient characteristics and differences tables.

**Additional file 2.**.Participant flow chart.

**Additional file 3.** Urban/rural status and food access interaction.

## Data Availability

The data that support the findings of this study are available from SPIROMICS but restrictions apply to the availability of these data, which were used under license for the current study, and so are not publicly available. Data are however available from the authors upon reasonable request and with permission of SPIROMICS. The datasets on food access generated and analysed during the current study are available in the Food Access Research Atlas repository, https://www.ers.usda.gov/data-products/food-access-research-atlas.aspx

## Abbreviations

COPD: Chronic obstructive pulmonary disease
SPIROMICS: SubPopulations and intermediate outcome measures in COPD study
FEV1%: Percentage of predicted forced expiratory volume in one second
SGRQ: St. George’s Respiratory Questionnaire
CAT: COPD assessment test
6MWD: 6-min walk distance test
mMRC: Modified medical research council
FARA: Food access research atlas
