# Supplementary material for: Disparities in access to food and chronic obstructive pulmonary disease (COPD)-related outcomes: a cross-sectional analysis
Source: BMC Pulm Med. 2021 Apr 27;21:139. doi: 10.1186/s12890-021-01485-8 (PMC8077917; doi:10.1186/s12890-021-01485-8)
Supplement: Supplementary file 1 — Additional file 1. Additional patient characteristics and differences tables. [file 12890_2021_1485_MOESM1_ESM.docx]

Disparities in access to food and Chronic Obstructive Pulmonary Disease (COPD)-related outcomes: A cross-sectional analysis.

Eric Moughames MD^1^, Han Woo PhD^1^ , Panagis Galiatsatos MD^1^, Karina Romero-Rivero MD^1^, Sarath Raju MD^1^, Vickram Tejwani MD^1^, Eric A. Hoffman PhD^2^, Alejandro P. Comellas MD^2^, Victor. E. Ortega MD^3^, Trisha Parekh DO^4^, Jerry. A. Krishnan MD^5^, Michael B. Drummond MD^6^, David Couper PhD^7^, Russell G. Buhr MD^8^, Robert Paine MD^9^, Joel. D. Kaufman MD^10^, Laura M. Paulin MD^11^, Nirupama Putcha MD^1^, Nadia. N. Hansel MD^1^.

**Affiliations:**
^1^Department of Medicine, Johns Hopkins University, Baltimore, MD, United States.

^2^Department of Internal Medicine, University of Iowa, Iowa City, IA, United States

^3^Department of Medicine, Wake Forest School of Medicine, Winston Salem, NC, United States.

^4^Department of Medicine, University of Alabama, Birmingham, AL, United States.

^5^Department of Medicine, University of Illinois, Chicago, IL, United States.

^6^Department of Medicine, University of North Carolina at Chapel Hill, Chapel Hill, NC, United States.

^7^Department of Biostatistics, University of North Carolina at Chapel Hill, Chapel Hill, NC, United States.

^8^Department of Medicine, Greater Los Angeles Veterans Affairs Healthcare System, Los Angeles, California, United States

^9^Department of Medicine, University of Utah, Salt Lake City, UT, United States.

^10^Department of Medicine and Epidemiology, University of Washington, Seattle, WA, Unites States.

^11^Department of Medicine, Dartmouth Hitchcock Medical Center, Lebanon, NH, United States.

**Appendix:**

**Appendix Table E1. Participant characteristics living in low food access areas.**

|  |  |  |  |
| --- | --- | --- | --- |
|  | **Low Food Access^1^ (N=1775)** | **Non-Low Food Access^1^ (N=938)** | **P-value** |
| COPD (Strata 3 & 4), % | 67.9 | 63.2 | **0.014** |
| FEV1 % pred., mean ± SD | 72.1 ± 26.6 | 74.8 ± 26.3 | **0.011** |
| Age, mean ± SD | 63.8 ± 8.7 | 63.0 ± 9.3 | **0.025** |
| Female, % | 46.1 | 45.9 | 0.946 |
| White, % | 79.9 | 70.4 | **<0.001** |
| Some college or above, % | 57.7 | 53.2 | **0.025** |
| Income, % |  |  | **<0.001** |
| Under $15,000, % | 17.0 | 26.5 |  |
| $15,000-$34,999, % | 19.3 | 18.7 |  |
| $35,000-$49,999, % | 13.2 | 11.1 |  |
| $50,000-$74,999, % | 16.2 | 10.6 |  |
| > $75,000, % | 19.4 | 12.0 |  |
| Decline to answer, % | 14.9 | 21.1 |  |
| Married, % | 50.9 | 38.9 | **<0.001** |
| Nonrural, % | 95.7 | 76.0 | **<0.001** |
| Body Mass Index, mean ± SD | 27.9 ± 5.27 | 27.9 ± 5.32 | 0.996 |
| Currently Smoking, % | 38.8 | 42.2 | 0.083 |
| Pack Years, mean ± SD | 49.3 ± 27.9 | 49.3 ± 25.1 | 0.953 |
| Low Income Tract, % ^2^ | 34.0 | 52.0 | **<0.001** |

Data are given as percentages unless otherwise indicated

^1^Low food access refers to a census tract with at least 500 people or 33 percent of the population living more than 1/2 mile (urban areas) or 10 miles (rural areas) from the nearest supermarket, supercenter, or large grocery store.

^1^Low income tract refers to a census tract where the tract's poverty rate is 20 percent or greater, or the tract's median family income is less than or equal to 80% of the State-wide median family income, or the tract is in a metropolitan area and has a median family income less than or equal to 80 percent of the metropolitan area's median family income.

^2^Stratum 3 includes participants with >20 pack-years and mild/moderate COPD with FEV_1_ /FVC <0.7 and FEV1>50% predicted (GOLD stage 1 and 2) measured during enrollment. Stratum 4 includes participants with >20 pack-years and severe COPD with FEV_1_ /FVC <0.7 and FEV_1_<50% predicted (GOLD stage 3 and 4) measured during enrollment^24^.

**Appendix Table E2. Differences (95% CI) in COPD-related outcomes for participants residing in food desert (vs. non-food desert), based on fully adjusted model, with additional adjustment by FEV1 % Predicted.**

|  |  |  |  |  |
| --- | --- | --- | --- | --- |
|  | Adjusted^1^ | | |  |
|  | *Mean Difference or odds ratio (95% CI)* | | *P*-Value |  |
| **Lung Function** |  |  |  |  |
| COPD (odds ratio) | 1.15 | (0.83, 1.59) | 0.404 |  |
| **Quality of Life/Respiratory Symptoms** |  |  |  |  |
| CAT | 0.88 | (0.15, 1.62) | **0.019** |  |
| mMRC | 0.02 | (-0.07, 0.11) | 0.685 |  |
| SGRQ Total | 2.32 | (0.61, 4.03) | **0.008** |  |
| 6-Minute Walk Distance (m) | -8.50 | (-18.7, 1.72) | 0.103 |  |
| **Chest CT Metric** |  |  |  |  |
| % Emphysema (-950) | -0.31 | (-1.02, 0.41) | 0.401 |  |
| % Air Trapping (-856) | 1.17 | (-0.11, 2.45) | **0.007** |  |
| **Any Exacerbation Event, last 12months.** |  |  |  |  |
| Any (OR)^2^ | 1.27 | (0.99, 1.62) | 0.055 |  |
| Severe (OR) | 1.46 | (1.07, 2.00) | **0.017** |  |

^1^The results for “All” participants are based on the fully adjusted main model with the addition of FEV1 % predicted as a covariate. ^2^Coefficient represents odds ratio.

**Abbreviations:**

6MWD: 6-minute walk distance test

CAT: COPD Assessment Test

COPD: Chronic Obstructive Pulmonary Disease

FEV1%: percentage of predicted forced expiratory volume in one second

mMRC: modified Medical Research Council

SGRQ: St. George’s Respiratory Questionnaire

**Appendix Table E3.** **Differences (95% CI) in COPD-related outcomes for former and current smokers in study population residing in food desert areas versus non-food desert areas by COPD status.**

|  |  |  |  |  |  |  |  |  | | |
| --- | --- | --- | --- | --- | --- | --- | --- | --- | --- | --- |
|  | Non-COPD (N=915) | | |  | COPD (N=1798) | | | |  |  |
|  | *Mean Difference or odds ratio (95% CI)* | | *P*-Val |  | *Mean Difference or odds ratio (95% CI)* | | *P*-Val | *p-*Val (Interaction) | | |
| **Lung Function** |  |  |  |  |  |  |  |  | | |
| FEV%Pred | 0.36 | (-1.95, 2.67) | 0.759 |  | -1.87 | (-4.50, 0.76) | 0.163 | 0.198 | | |
| **Quality of Life/Respiratory Symptoms** |  |  |  |  |  |  |  |  | | |
| CAT | 0.98 | (-0.36, 2.31) | 0.151 |  | 0.99 | (0.04, 1.95) | **0.042** | 0.898 | | |
| mMRC | 0.06 | (-0.09, 0.21) | 0.412 |  | 0.03 | (-0.09, 0.15) | 0.636 | 0.920 | | |
| SGRQ Total | 2.04 | (-1.23, 5.30) | 0.222 |  | 2.76 | (0.52, 5.01) | **0.016** | 0.416 | | |
| 6-Minute Walk Distance (meters) | -0.88 | (-15.6, 13.8) | 0.906 |  | -16.8 | (-31.4, -2.16) | **0.025** | 0.055 | | |
| **Chest CT Metric** |  |  |  |  |  |  |  |  | | |
| % Emphysema (-950) | -0.06 | (-0.34, 0.23) | 0.707 |  | 0.09 | (-1.09, 1.26) | 0.882 | 0.337 | | |
| % Air Trapping (-856) | 0.98 | (-0.70, 2.66) | 0.251 |  | 2.42 | (0.23, 4.61) | **0.030** | 0.861 | | |
| **Exacerbations, last 12months** |  |  |  |  |  |  |  |  | | |
| Any (Odds Ratio) | 0.99 | (0.58, 1.67) | 0.960 |  | 1.47 | (1.12, 1.93) | **0.006** | 0.129 | | |
| Severe (Odds Ratio) | 1.36 | (0.64, 2.89) | 0.422 |  | 1.59 | (1.13, 2.23) | **0.008** | 0.701 | | |
|  |  |  |  |  |  |  |  |  | | |

^‡^Coefficient represents odds ratio.

**Abbreviations:**

6MWD: 6-minute walk distance test

CAT: COPD Assessment Test

COPD: Chronic Obstructive Pulmonary Disease

FEV1%: percentage of predicted forced expiratory volume in one second

mMRC: modified Medical Research Council

SGRQ: St. George’s Respiratory Questionnaire
