# Supplementary material for: Disparities in access to food and chronic obstructive pulmonary disease (COPD)-related outcomes: a cross-sectional analysis
Source: BMC Pulm Med. 2021 Apr 27;21:139. doi: 10.1186/s12890-021-01485-8 (PMC8077917; doi:10.1186/s12890-021-01485-8)
Supplement: Supplementary file 2 — Additional file 2..Participant flow chart. [file 12890_2021_1485_MOESM2_ESM.docx]

**Appendix Figure E1**. **Participant flow chart**

Participant flowchart indicating the number of SubPopulations and InteRmediate Outcome Measures (SPIROMICS) Air participants. 68 participants were excluded due to missing census geographical identifiers, food desert data or did not provide consent for geocoding. 201 participants who are healthy controls were also excluded.
