# Supplementary material for: Disparities in access to food and chronic obstructive pulmonary disease (COPD)-related outcomes: a cross-sectional analysis
Source: BMC Pulm Med. 2021 Apr 27;21:139. doi: 10.1186/s12890-021-01485-8 (PMC8077917; doi:10.1186/s12890-021-01485-8)
Supplement: Supplementary file 3 — Additional file 3. Urban/rural status and food access interaction. [file 12890_2021_1485_MOESM3_ESM.docx]

**Appendix Figure E2. Adverse Association of Limited Food Access is Greater in Urban Areas**

The chart shows the urban/rural x food access interaction effect estimate and its 95% confidence interval or, equivalently, the urban-rural difference in food access’ association with COPD outcomes (or, the urban-rural difference in the mean difference in COPD outcome by food access), adjusting for neighborhood poverty, clinical centers, age, sex, race, education, income, marital status, rural status, BMI, smoking status, and pack years.

^a^ For the dichotomous outcomes—COPD, any exacerbation, and severe exacerbation, the urban-rural difference represents the log odds, multiplied by 10 to fit the chart’s scale.

^b^ For mMRC, 6MWD, the point estimate and its 95% confidence interval were rescaled to fit the chart: multiplied by 10 for mMRC, divided by 10 for 6MWD.
